# Amphipathic Octenyl‐Alanine Modified Peptides Mediate Effective siRNA Delivery

**DOI:** 10.1002/psc.70054

**Published:** 2025-09-07

**Authors:** Tõnis Lehto, Marit Isakannu, Helena Sork, Annely Lorents, Safa Bazaz, Oscar P. B. Wiklander, Samir EL Andaloussi, Taavi Lehto

**Affiliations:** ^1^ Institute of Technology, University of Tartu Tartu Estonia; ^2^ Faculty of Medicine, Institute of Pharmacy University of Tartu Tartu Estonia; ^3^ Division for Biomolecular and Cellular Medicine, Department of Laboratory Medicine Karolinska Institute Stockholm Sweden; ^4^ Center for Hematology and Regenerative Medicine (HERM), Department of Medicine Huddinge Karolinska Institute Stockholm Sweden; ^5^ Center of Research and Strategic Studies Lebanese French University Erbil Iraq

**Keywords:** cell‐penetrating peptide, drug delivery, hydrophobically modified peptides, oligonucleotide delivery, RNAi

## Abstract

The development of therapeutic small interfering RNAs (siRNAs) has lately gained significant momentum due to their ability to silence genes in a highly specific manner. The main obstacle withholding the wider translation of siRNA‐based drug modalities is their limited half‐life and poor bioavailability, especially in extra‐hepatic tissues. Consequently, various drug delivery systems (DDSs) have been developed to improve the delivery of siRNAs, including short delivery peptides called cell‐penetrating peptides (CPPs). In this study, we explore the potential of using alkenyl‐alanine modifications to enhance the siRNA delivery efficacy with CPPs. We demonstrate on hPep peptides that incorporation of alkenyl‐alanines enhances the encapsulation of siRNAs into stable nanoparticles and contributes to increased cellular uptake. Furthermore, we demonstrate that the lead peptide, hPep3, induces effective RNAi‐mediated gene silencing in a reporter cell model as well as on the disease‐implicated endogenous CD45 gene target. The biodistribution studies in mice show that the alkenyl‐alanines are systemically well tolerated, and employing such modifications in the peptide backbone improves siRNA delivery in several tissues, including extra‐hepatic sites. As demonstrated on hPep peptides, alkenyl‐alanines offer a simple yet robust way to enhance the delivery efficacy of CPPs and have the potential to advance siRNA therapeutics beyond the liver targets.

## Graphical abstract

This study demonstrates that novel octenyl‐alanine–modified hPep peptides efficiently encapsulate siRNA into well‐defined nanoparticles. Our findings highlight the potential of these hPep/siRNA nanoparticles to induce RNA interference, effectively silencing therapeutically important CD45 expression.

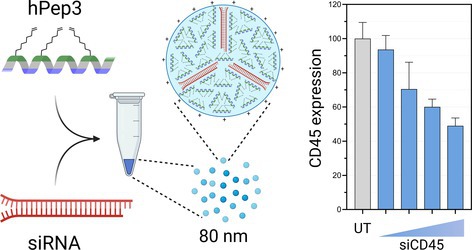

## 1 Introduction

RNA interference (RNAi) therapeutics, which are based on short interfering RNAs (siRNAs), carry enormous potential for the treatment of human diseases [1]. The unique feature of siRNAs is their ability to selectively silence any gene by targeting its expression at the mRNA level via the endogenous post‐transcriptional gene silencing mechanism of RNAi [2]. The therapeutic potential of siRNAs has lately been demonstrated by numerous preclinical and clinical studies targeting various diseases, including various cancers, viral infections, metabolic, autoimmune, and neurological disorders, to name a few [3].

Even though siRNAs possess excellent therapeutic potential, there are several hurdles that hamper the wider translation of RNAi therapeutics into the clinics. These include inherently high molecular weight (~13–17 kDa) and negative net charge of siRNAs that severely limit their ability to spontaneously cross cellular membranes and consequently exert biological activity inside the cells. The half‐life/bioavailability of siRNAs is further affected by their proneness to degradation by various extracellular and intracellular nucleases, rapid renal clearance, and propensity of being detected by pattern recognition receptors of the immune system [1, 3]. To a large extent, the issues with stability and immunogenicity have been overcome by chemical engineering of the siRNAs. Successful approaches include the incorporation of phosphorothioate linkages in the terminal ends of both the guide and the passenger strand and optimized positioning of 2′‐*O*‐methyl and 2′‐deoxy‐2′‐fluoro substitutions at selected bases, providing higher overall nuclease stability [4, 5]. Though these modifications have drastically improved the potency of siRNAs by enhancing enzymatic stability and lowering immunogenicity, high renal clearance and low cellular uptake efficacy still greatly limit their bioavailability. Therefore, further clinical translation of siRNA therapeutics requires the development of effective drug delivery technologies.

A broad range of drug delivery systems (DDSs) have been studied for the delivery of siRNAs, with main strategies including encapsulation of the siRNA molecules into nanoparticles (NPs) or generating siRNA bioconjugates with bioavailability‐enhancing modifications such as receptor ligands or fatty acids [6, 7, 8]. Among these approaches, lipid nanoparticles (LNPs) and bioconjugates have been the most successful in clinical development, as exemplified by the first‐in‐class market authorization of LNP‐based formulation Onpattro (patisiran) and GalNAc‐siRNA conjugate Gilvaari (givosiran) in recent years. Since both platforms are almost exclusively active in the liver, particularly in hepatocytes, main efforts in clinical development have naturally focused on various liver diseases. Beyond the liver, effective drug delivery means are lacking, hindering the wider application of siRNA therapeutics.

To tackle this, various types of delivery modalities such as cationic polymers [9], dendrimers [10] and peptides [11], including cell‐penetrating peptides (CPPs), are actively studied. CPPs are generally short (up to 30 amino acids long) cationic and/or amphipathic peptides that can condense various nucleic acid‐based molecules into stable NPs via electrostatic, hydrophobic, and other intermolecular interactions [11]. Owing to their capability to deliver different molecules across cellular membranes [11, 12, 13], CPPs represent attractive means for the delivery of siRNA.

In the context of developing peptide‐based delivery vectors, it has been observed that classical cationic CPPs such as Tat and oligoarginines tend to have limited delivery efficacy for oligonucleotides (ON). Therefore, strategies such as fine‐tuning the peptide secondary structure and/or amphipathicity [14, 15], conjugating N‐terminal hydrophobic moieties [16, 17, 18, 19, 20, 21] incorporating unnatural amino acids [18, 22, 23] and applying endosomolytic modifications [24] represent a selection of approaches to improve the delivery potency of CPPs. Among those strategies, hydrophobic modifications significantly improve the delivery efficacy by enhancing the ability of CPPs to formulate stable CPP/ON nanocomplexes, interact with cell membrane lipids, increase cellular uptake, and modulate endosomal release [16, 19, 20, 21]. As an alternative, the hydrophobicity of CPPs can be promoted through structural modifications, including the incorporation of specific amino acid analogs, such as alkenyl‐alanines, which are commonly used for stapling [25]. This approach allows distributing hydrophobicity evenly across the peptide sequence. Also, the enhancement of the alpha‐helical folding that the alkenyl‐alanines provide is a commonly used strategy for creating biologically active stapled peptides for nucleic acid delivery.

Here, we demonstrate the potential of using alkenyl‐alanine modifications for the delivery of siRNA. By varying the number and length of alkenyl‐alanines, we show that the developed hPep peptides with different hydrophobicities can form stable NPs with siRNA and display efficient cellular uptake. Also, we show the importance of such modifications in peptide secondary structure, mediating membrane activity, facilitating efficient endosomal release, and promoting gene silencing. The accumulation of hPep/siRNA NPs in extra‐hepatic tissues in vivo demonstrates eloquently the proof‐of‐concept in employing alkenyl‐alanine modifications for targeting organs outside the liver.

## 2 Materials and Methods

### 2.1 CPPs

All the peptides used in the study (see Table 1) were ordered from Pepscan Presto (now Biosynth) (Lelystad, the Netherlands) with > 90% purity specification based on ultraperformance liquid chromatography. The peptides are C‐terminally amidated and have a free amine in the N‐terminus. All peptides carry six positive charges at physiological pH.

**TABLE 1:** The sequences of used peptides.

Name	Sequence	Mw (g/mol)
hPep1	H‐LAKLAKA{R8}AKLLKA{S5}AKAL‐NH_2_	2040.4
hPep2	H‐LAKLAKA{R8}AKLLKA{S8}AKAL‐NH_2_	2084.4
hPep3	H‐L{R8}KLAKA{R8}AKLLKA{S8}AKAL‐NH_2_	2193.9
Ctrl	H‐LAKLAKAAAKLLKAAAKAL‐NH_2_	1863.4

*Note:* R8 stands for (*R*)‐2‐(7‐octenyl)alanine; S8 stands for (*S*)‐2‐(7‐octenyl)alanine, and S5 stands for (*S*)‐2‐(4‐pentenyl)alanine.

### 2.2 Oligonucleotides

siRNAs used in the study were ordered from Integrated DNA Technologies (USA) unless stated otherwise. The siRNAs used were against luciferase (siLuc), CD45 (siCD45) (Axolabs, Germany), and an inactive control siRNA (siCTRL). The sequences of the guide (antisense) and passenger (sense) strands are shown in Table 2.

**TABLE 2:** The sequences of used siRNAs.

Name		Sequence
siLuc	Sense strand	5′‐UUCUUUAUGUUUUUGGCGU**CU**‐3′
	Antisense strand	3′‐**GA**AAGAAAUACAAAAACCGCA‐5′
siLuc2	Sense strand	5′‐GGACGAGGACGAGCACUUC**UU**‐3′
	Antisense strand	3′‐**UU**CCUGCUCCUGCUCGUGAAG‐5′
siCTRL	Sense strand	5′‐CCGUGUGAAUCAUUGUCUU‐3′
	Antisense strand	3′‐GGCACACUUAGUAACAGAC‐5′
siCD45	Sense strand	5′‐cuGGcuGAAuuucAGAGcATsT‐3′
	Antisense strand	5′‐UGCUCUGAAAUUcAGCcAGTsT‐3′

*Note:* The markings in bold show the dinucleotide overhang. N: RNA residues, n: 2′‐*O*‐methyl residues, s: phosphorothioate backbone modification.

### 2.3 Circular Dichroism (CD) Spectroscopy

CD spectra were recorded on a Jasco J‐1500 CD spectrometer (Jasco, Japan) at 25°C at a concentration of 100 μM. Peptides were dissolved in MQ water and measured using 1‐mm pathlength quartz cuvette. The secondary structure estimates were calculated on the “Protein Secondary Structure Estimation” plugin in the Spectra Manager 2.13 software by using Reed's estimation.

### 2.4 Noncovalent Peptide/siRNA Complex Formulation

Peptide/siRNA complexes were prepared by using the non‐covalent formulation method. Complexes were prepared via pipette mixing at peptide‐to‐siRNA molar ratios (MRs) 0, 5, 10, 15, 20, 25, 30, and 35, which correspond to N/P ratios 0, 0.7, 1.4, 2.1, 2.9, 3.6, 4.3, and 5.0, respectively. Working solutions of 100 μM peptide and 10 μM siRNA diluted in MQ were used to mix in vitro complexes, while 1 mM peptide and 100 μM siRNA working solutions were used for in vivo complexes. Based on the specific experiment, the complexes were prepared either in MQ, HEPES 7.4 buffer (Fisher Scientific, USA), or HEPES‐buffered glucose HBG, 20 mM HEPES, pH = 7.4, 5% glucose (Fisher Scientific, USA). After formulation, complexes were let to stabilize by incubating for 30 min at RT.

### 2.5 SYBR Gold Exclusion Assay

For SYBR Gold exclusion assay, peptide/siRNA complexes were formed in 20 mM HEPES 7.4 solution at MRs 0–35, as stated above. Twenty microliters of complexes were mixed with 100 μL of 2X SYBR Gold dye (Thermo Fisher Scientific, USA) and 80 μL of 20 mM HEPES 7.4 buffer, leading to a final siRNA concentration of 100 nM. The mixture was transferred to black 96‐well plates and incubated for 15 min at RT. The experiment used Synergy Mx Microplate Reader (BioTek, USA) (ex = 495 nm, em = 537 nm) to determine the amount of accessible siRNA in the complexes. The data were normalized to the fluorescence of free siRNA (defined as 100%) and are expressed as a percentage of encapsulation efficiency. The results are shown as mean ± SEM of *n* = 3 in duplicates.

### 2.6 Heparin Displacement Assay

For the heparin displacement assay, peptide/siRNA complexes were formed in MQ water at MR30. Fifty microliters of complexes was then transferred to each well of a black 96‐well plate and mixed with 100 μL of 2X SYBR Gold dye (Thermo Fisher Scientific, USA), incubated, and then 50 μL of heparin (Sigma‐Aldrich, USA) solution with different concentrations was added. After 15 min at RT, the fluorescence was measured by Synergy Mx Microplate Reader (BioTek, USA) (ex = 495 nm, em = 537 nm) to determine the amount of accessible siRNA in the complexes. The data was normalized to the fluorescence of free siRNA (defined as 100%) and are expressed as a percentage of accessible siRNA. The results are shown as mean ± SEM of *n* = 3 in duplicates.

### 2.7 Physicochemical Characterization of hPep/siRNA Complexes

The physicochemical properties (nanoparticle size, polydispersity index [PDI], and zeta potential) of peptide/siLuc complexes were measured by DLS (dynamic light scattering) using the Zetasizer Nano ZS apparatus (Malvern Instruments, UK).

The size and zeta potential of peptide/siLuc particles were measured at MR30 for hPep1, hPep2, and Ctrl peptide. For hPep3, measurements were also taken at different MRs (5–35). The complexes were formulated in 50 μL at a 2 μM final siRNA concentration for size measurement. The average diameter was obtained from three technical replicates, and one measurement was set to five runs (run duration 8 s) in disposable ZEN0040 cuvettes (Malvern Instruments, UK).

To measure the zeta potential, the complexes were diluted eight times in 10 mM HEPES 7.4 buffer. The zeta potential was obtained from three technical measurements in disposable DTS1070 cuvettes (Malvern Instruments, UK) with the number of runs set to automatic. The results are shown as an average of three separate experiments (mean ± SEM). All measurements were performed at 22°C.

### 2.8 Hemolysis Assay

The hemolysis experiments were carried out with bovine red blood cells (RBCs) (Håtunalab, Sweden). The cells were washed three times with HBG 7.4 and then diluted to a final concentration of 2% RBCs in HBG with noted pH. Ten microliters of peptides was added to 190 μL of cells in PCR plates and shaken at 37°C for 1 h. Then the cells were spun down at 500 × *g*, and 100 μL of supernatant was transferred to transparent 96‐well tissue culture plates. The absorbance of released hemoglobin was measured at 450 nm on a plate reader (Tecan Sunrise, Switzerland). As a positive control, 10% Triton X‐100 was used, while HBG served as a negative control. The percentage of hemolysis was calculated by the formula: hemolysis (%) = [(A_S_ − A_N_)/(A_P_ − A_N_)] × 100, where A_S_ is the absorbance for the sample, A_P_ for positive control, and A_N_ for negative control. The results are shown as mean ± SEM of *n* = 3, done in triplicates.

### 2.9 Cell Culture

For the in vitro experiments, U87 and HEK‐293 cells stably expressing firefly luciferase (U87‐Luc2 and HEK‐Luc) were used. Both cell lines were cultivated in DMEM (Dulbecco's Modified Eagle's Medium, Thermo Fisher Scientific, USA) containing 100 U/mL penicillin (Sigma‐Aldrich, USA), 100 μg/mL streptomycin (Thermo Fisher Scientific, USA), and 10% FBS (fetal bovine serum; Thermo Fisher Scientific, USA). The cells were maintained in a water‐jacketed incubator at 37°C, 5% CO_2_ atmosphere, and split every 2–3 days.

### 2.10 Quantitative Uptake

For cellular uptake studies, 20,000 cells per well were seeded in 96‐well cell culture plates. After 24 h, cells were treated with either peptide/AlexaFluor568‐siRNA complexes at MR30 or AlexaFluor568‐siRNA (AF568‐siRNA) as a control. The complexes were added to the cells in 1/10 of the final volume of the cell media (100 μL) at 12.5–200 nM final siRNA concentration and incubated for 4 h at 37°C. To remove residual complexes from the extracellular environment, the cells were washed two times with 100 μg/mL cold heparin solution in DPBS (Thermo Fisher Scientific, USA). To release the complexes from the intracellular milieu, the cells were lysed with 0.1% Triton X‐100 solution (Sigma‐Aldrich, Germany) in DPBS (Thermo Fisher Scientific, USA). The fluorescence from the complexes was measured with a Synergy Mx Microplate Reader (BioTek, USA) (λ_ex_ = 568 nm, λ_em_ = 603 nm) in black 96‐well plates containing 45 μL of cell lysate and 105 μL of DPBS solution. The values were normalized to the protein content (Bio‐Rad DC Protein Assay, Bio‐Rad, USA) and are shown as RFU/μg of total protein. Three independent experiments were carried out in duplicates, and the results are shown as mean ± SEM.

### 2.11 Confocal Laser Scanning Microscopy

For microscopy experiments, 50,000 U87‐Luc2 cells per well were seeded on round glass coverslips (VWR, Germany) in 24‐well cell culture plates. After 48 h, cells were treated with peptide/AF568‐siRNA complexes at MR30 or AF568‐siRNA alone. Complexes were added to the cells in 1/10 of the final volume of cell media at a 50 nM final siRNA concentration, and cells were incubated for 1 or 4 h at 37°C. Treated cells were washed twice with PBS and fixed with 4% paraformaldehyde in 0.1 M PBS (pH 7.4) for 30 min at room temperature. Fixed cells were washed twice with PBS, and nuclei of cells were stained with Hoechst 33342 solution (1 μg/mL in PBS) (Thermo Fischer Scientific, USA) for 5 min at room temperature. The coverslips were mounted on glass slides with 50% glycerol (in PBS) and analyzed with a Zeiss LSM900 confocal microscope (Carl Zeiss, Germany).

### 2.12 Luciferase Knockdown in HEK‐Luc Cells

Gene knockdown efficacy of the peptides was evaluated by treating the HEK‐Luc luciferase reporter cell line with hPep/siRNA complexes. Twenty‐four hours before treatment, 7000 cells per well were seeded in 96‐well plates. The siRNA/peptide complexes formulated at MR30 with siLuc or siCTRL were added to the cells in 1/10 of the final volume of the cell media (100 μL) at final treatment concentrations of 12.5–200 nM. After 24 h treatment at 37°C, the cells were lysed with 50 μL of 0.1% Triton X‐100 solution in DPBS. For the luciferase activity measurement, 40 μL of LAR buffer solution (20 mM glycylglycine, 1 mM MgCl_2_, 0.1 mM EDTA, 3.29 mM DTT, 0.548 mM adenosine 5′‐triphosphate, and 0.0013 mM coenzyme A; pH 8–8.5) supplemented with 5% (v/v) of a mixture of 10 mM luciferin and 29.375 mM glycylglycine was added to the lysate. The luminescence was measured with a GLOMAX luminometer (Promega Corporation, Sweden) and the obtained RLU (relative light unit) values were normalized to the RLU value of untreated cells. The results are shown as a percentage of remaining gene expression using mean ± SEM of *n* = 3, performed in duplicates.

For chloroquine (CQ)‐induced endosomal release, the cells were treated for 4 h at 37°C with hPep3/siRNA complexes at 200 nM siRNA concentration (MR30) in serum‐containing DMEM. This was followed by the addition of 50 μM CQ (Sigma‐Aldrich, USA) for 2 h, after which the media was changed to fresh serum‐containing media. Finally, the luciferase expression was measured after 18 h of incubation. The results are shown as mean ± SEM of *n* = 3, done in duplicates.

### 2.13 WST‐1 Cell Viability Assay

To evaluate the possible impact of peptide/siRNA NPs on cell viability, the WST‐1 assay (Roche, Germany) was used according to the manufacturer's protocol. Twenty‐four hours before treatment, 7000 cells per well were seeded in 96‐well plates. The cells were treated with peptide/siLuc complexes at the final siRNA concentrations of 12.5–200 nM (MR30) or with siLuc/Lipofectamine 2000 (LF2000, Thermo Fisher Scientific, USA) complexes formed according to the manufacturer's instructions in OptiMEM (Thermo Fisher Scientific, USA). The complexes were added to the cells in 1/10 of the final volume of the cell media (100 μL). As a positive control, DMSO (Sigma‐Aldrich, Germany) was used. Twenty‐four hours after the treatment, 10 μL of WST‐1 reagent was added per well and incubated for another 3 h, followed by absorbance measurement at 450/690 nm with Sunrise absorbance plate reader (Tecan Trading AG, Switzerland). The results are shown as mean ± SEM of *n* = 3, done in duplicates.

### 2.14 Knockdown of Endogenous Target (CD45)

The knockdown of an endogenous CD45 target was evaluated in the murine macrophage cell line Raw 264.7. Briefly, 50,000 cells per well were seeded in 24‐well plates 24 h before treating them with siCD45/hPep3 or siCTRL/hPep3 nanocomplexes formed at MR30 (at a final siRNA concentration of 50–200 nM) or with Lipofectamine 2000 (LF2000, Thermo Fisher Scientific, USA; at a concentration of 3.25–25 nM). After 24 h, the cells were collected by trypsinization, washed once with DPBS (Thermo Fisher Scientific, USA), and stained using LIVE/DEAD Fixable Near‐IR Dead Cell Stain Kit (# L34975, Invitrogen, Thermo Fisher Scientific, USA) according to manufacturers' recommendations. After that, the cells were subjected to surface staining with APC anti‐mouse CD45 (Cat. No. 103112; 1:100 dilution) or APC Rat IgG2b, κ Isotype Ctrl (Cat. No. 400612; 1:100 dilution) (both BioLegend, USA) antibodies. After the incubation on ice in the dark for 20 min, the cells were washed with DPBS (Thermo Fisher Scientific, USA), resuspended in FACS buffer (DPBS, 2% FCS, 1 mM EDTA), and analyzed on the Attune NxT Flow Cytometer (Thermo Fisher Scientific, USA). Data analysis was performed with FlowJo 10.8.1 software, followed by visualization using GraphPad Prism (Version 9). The results are expressed as mean ± SEM of *n* = 3 experiments.

### 2.15 Animal Study

For the in vivo biodistribution studies, 20‐g female BALB/c mice were injected intravenously with AF568‐siRNA and hPep3/AF568‐siRNA complexes formulated at MR30 in HBG with a total of 20 μg of AF568‐siRNA per animal (three animals per group). Four hours after the treatment, the animals were euthanized, and organs harvested. The biodistribution of the hPep3/AF568‐siRNA NPs was evaluated on the homogenized tissues via fluorescence quantification. Briefly, intact tissues were placed in T‐PER Tissue Protein Extraction Reagent (Thermo Fisher Scientific, USA) and subjected to homogenization using the Precellys 24 dual homogenization system (Bertin Technologies, France). The homogenate was centrifuged for 5 min at 10,000 × *g*, and the supernatant was employed to gain a fluorescence measurement on a Synergy Mx Microplate Reader (BioTek, USA). The fluorescence values were normalized against Bio‐Rad DC Protein Assay (Bio‐Rad, USA) total protein measurements and are expressed as RLU/μg of total protein. The animal experiments were carried out in accordance with the Guidelines for Care and Use of Laboratory Animals of the University of Tartu and were approved by the Estonian Laboratory Animal Ethics Committee (Approval No. 185, dated February 16, 2021).

### 2.16 Statistical Analysis

Data are presented as mean with a standard error of the (mean ± SEM) of at least three independent experiments. Significant differences were evaluated by analysis of variance (ANOVA) with Dunnett's multiple comparisons test unless stated otherwise (GraphPad Prism 9; GraphPad Software Inc., San Diego, CA, USA). In all cases, differences with *p* < 0.05 were deemed to be significant (*p < 0.05, **p < 0.01, ***p < 0.001, and ****p < 0.0001).

## 3 Results and Discussion

### 3.1 Characterization of hPep Peptides and hPep/siRNA Complex Formation

**FIGURE 1:**
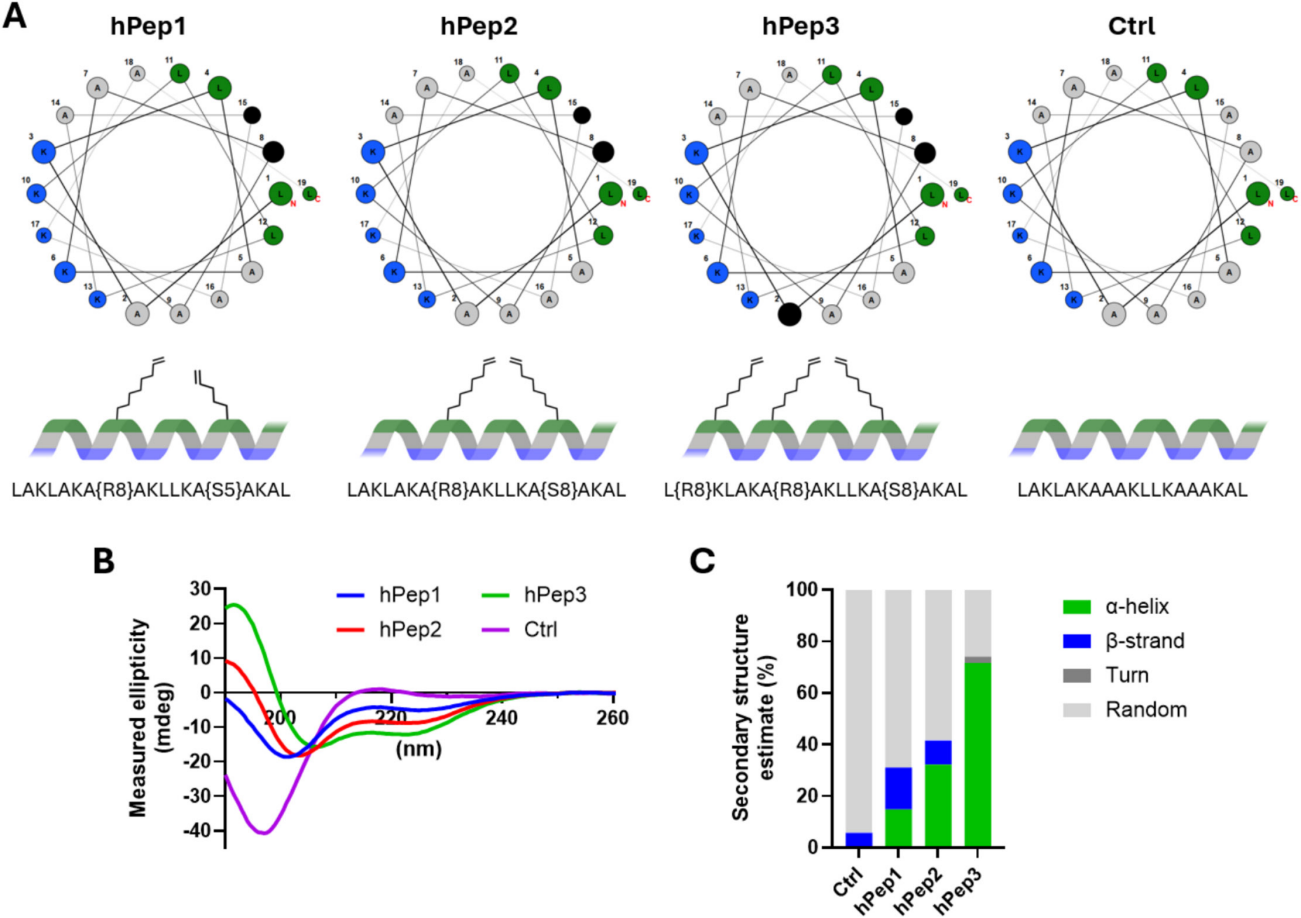
Structural properties of hPep peptides. (A) Helical wheel projections of hPep peptides were produced with https://clemlab.github.io/helicalwheel/. In the projections cationic amino acids (blue) are separated from hydrophobic (green) and modified (black) amino acids by neutral alanines (gray). The twisted ribbon helices below illustrate the structure and positioning of the modifications along the helix axis. All peptides presented above carry charge of +6 at physiological pH. (B) To evaluate the secondary structure of hPep peptides the CD was measured in water at 100 μM peptide. (C) The secondary structure estimates were calculated on the onboard “Protein Secondary Structure Estimation” software by using Reed's estimation.

Positively charged CPPs, like other cationic delivery systems, are known to interact with negatively charged oligonucleotides and form complexes. Among those, the hPep family of peptides has recently gained attention [22, 26]. hPep peptides are designed around the idea of an ideal alpha helical peptide where hydrophobic leucines on one facet of the helix are separated from cationic lysines on the other side of the helix by alanines. By incorporating different alkenyl‐alanines into the hydrophobic side of the helix, it is possible to increase the hydrophobic moment and amphipathicity of the peptides. In hPep1, we incorporated octenyl‐alanine at Position 8 and pentenyl‐alanine at Position 15, while in hPep2, octenyl‐alanines were utilized in both positions. In hPep3, we incorporated a third octenyl‐alanine at Position 2 to enhance the hydrophobic character of the peptide even further. The Ctrl peptide resembles hPep3 but with natural alanines instead of alkenyl‐alanines (Table 1 and Figure 1A). When we estimated the secondary structure of the peptides with CD, we found that the proportion of alpha helical structure started to increase from zero for the Ctrl peptide with the addition of longer and more alkenyl‐alanines to 15% for hPep1 and 32% for hPep2 (Figure 1B,C). hPep3, with the three octenyl‐alanines, reached even up to 70% in helical content, confirming that hPep3 is a strongly helical and amphipathic peptide. On the other hand, hPep1, hPep2, and the Ctrl peptide were mainly unstructured, as indicated by the high random structure estimates (> 60%), while also small portions of β‐stranded structures were noted. Therefore, in Figure 1A, the helical wheel projections for hPep1, hPep2, and the Ctrl peptide should be taken with a small caveat, as they are meant to illustrate the design principles of hPep peptides and not their actual secondary structure.

**FIGURE 2:**
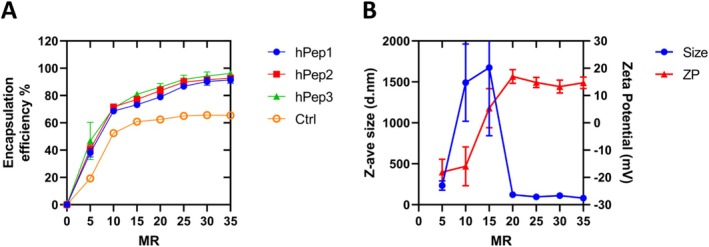
Characterization of complex formation between hPeps and siRNA. (A) Peptide/siRNA complexes were formed at different MRs and show an enhanced encapsulation efficiency (EE%) at higher MRs. Encapsulation efficiency was measured with SYBR Gold exclusion assay, and the results were normalized to the signal of free siRNA and transformed to EE%. (B) The average hydrodynamic diameter (d.nm) and zeta potential (mV) of hPep3/siRNA NPs at different MRs were measured by DLS. The values represent the mean of at least three independent experiments (mean ± SEM, *n* = 3).

To understand the capacity of hPep peptides to form complexes with siRNA, we set out to study the siRNA encapsulation efficiency (EE%) at various peptide/siRNA MRs ranging from zero to 35 by using a SYBR Gold exclusion assay. In this assay, pre‐formed complexes are exposed to a nucleic acid‐binding dye, which allows us to monitor the siRNA encapsulation efficiency by fluorescence spectroscopy. The tested MRs (0–35) translate to N/P ratios from zero to five (also termed charge ratio), where N stands for the number of positively charged peptide amines and P stands for negatively charged siRNA phosphates. Encapsulation data indicated that all the hPeps have a very similar siRNA encapsulation efficiency profile (Figure 2A). Roughly at MR7 (N/P ratio ~1.0), approximately 50% of the siRNA was encapsulated for all the hPeps. In contrast, the control peptide (Ctrl), carrying natural alanines instead of alkenyl‐alanines, had a slightly lower EE% of ~30%–40%. By further increasing the MR to MR30 (N/P ratio 4.3) and above, ≥ 90% siRNA encapsulation efficiency could be achieved with all the hPeps, while the Ctrl peptide had about 30% lower EE% (~60%) at MR30 (Figure 2A). The lower EE% of the Ctrl peptide emphasizes the important role of alkenyl‐alanines in promoting efficient complex formation with the hPep peptides while their differences in secondary structure were not found to affect the EE%.

We next wanted to understand how the peptide/siRNA MR influences the physicochemical properties of the complexes, and for this, we carried out DLS analysis to measure both the size and zeta potential of different hPep/siRNA complexes. The DLS data in Figure 2B illustrate the peptide amount dependent formation of hPep3/siRNA complexes where the zeta potential rises from −20 mV at MR5 to neutral at around MR15 (N/P ratio ~2.0). As such, the peptide positive charges have neutralized all the siRNA negative charges. Consequently, the lack of electrostatic repulsive forces between the neutrally charged particles leads to aggregation (NP size > 1000 nm). When the ratio increases to MR20 and above, stable NPs are formed with zeta potentials around +15 mV and sizes around 80–120 nm. Hence, having enough peptide during the complex formation is the key to achieving a nanosized peptide/siRNA formulation. Similar behavior was also found for peptides hPep1 and hPep2 (Figure S1A–C). On the other hand, with the Ctrl peptide, the zeta potential of the complexes stayed negative at all tested MRs (Figure S1D). This is likely due to its inability to sufficiently condense siRNA into NPs, as confirmed by DLS (Figure S1D) and encapsulation efficiency studies (Figure 2A), thus further emphasizing the importance of alkenyl modifications in efficient complex formation.

Based on these findings, we chose MR30 (N/P ratio 4.3) for further characterization studies as these conditions provide maximal encapsulation efficiency and stable nanoparticle formation.

### 3.2 Role of Alkenyl‐Alanines in the Formulation of Stable hPep/siRNA NPs

**FIGURE 3:**
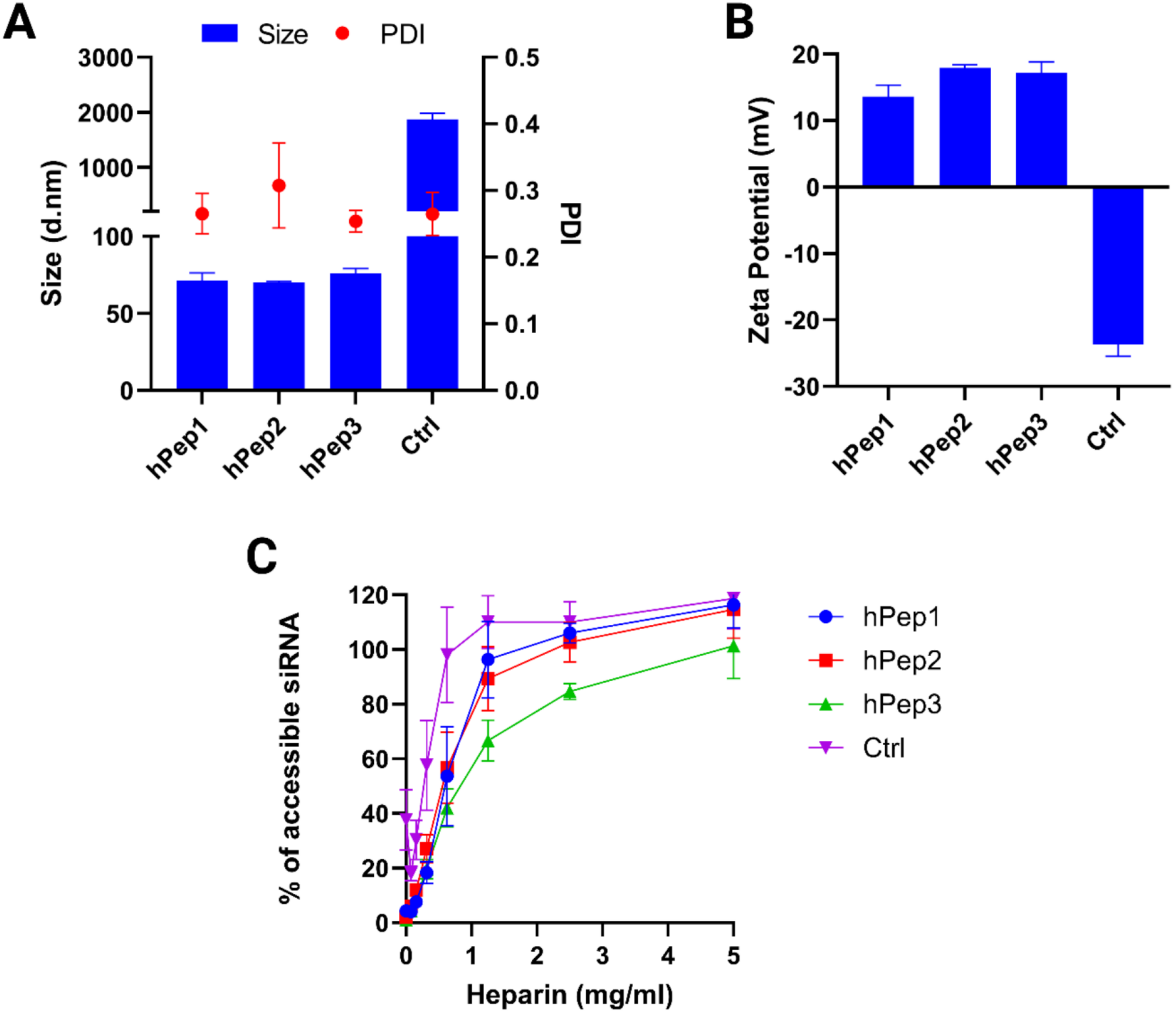
Physicochemical properties of hPep/siRNA NPs at MR30. (A) DLS was used to measure the size (blue), PDI (red), and (B) zeta potential of hPep/siRNA NPs formulated at MR30. (C) Heparin displacement assay was used to measure the stability of complexes. The values represent the mean of at least three independent experiments (mean ± SEM, *n* = 3).

After determining the optimal conditions for nanoparticle formation, we next set out to perform detailed physicochemical characterization of the peptide/siRNA NPs with all three hPeps. For that, the complexes were formulated at MR30. The size data from the DLS show all hPeps form homogeneous NPs in a very narrow size range of 70–80 nm with PDIs of 0.265, 0.308, and 0.254 for hPep1, hPep2, and hPep3, respectively (Figures 3A and S2). As shown in Figure 3B, hPep/siRNA NPs have positive surface charge with zeta potentials ranging from +13 to +18 mV for all hPeps. Meanwhile, the Ctrl peptide failed to encapsulate siRNA into NPs, forming unstable aggregates (~2000 nm) with loose structure (insufficient encapsulation efficiency) where anionic siRNA molecules can be exposed to the surface of the complexes; thus, causing the negative zeta potential (−23.7 mV) (Figure 3A,B).

It is known that hydrophobic interactions play a key role in the colloidal and enzymatic stability of peptide/nucleic acid NPs [19, 20]. Since the physicochemical characteristics of all the hPep/siRNA NPs were relatively similar, it is possible to deduce how the hydrophobic alkenyl modifications of different hPeps affect the stability of their respective complexes with siRNA. To characterize that, we used a heparin displacement assay. In this assay, complexes are exposed to different concentrations of heparin, and the differences in the release of siRNA are monitored by the SYBR Gold assay. The more heparin it takes to release the siRNA from the complexes, the more stable the interactions within the peptide/siRNA NPs are. Figure 3C shows that the stability of the NPs increases in the order of hydrophobicity of the peptides Ctrl < hPep1 < hPep2 < hPep3 [22]. Furthermore, it can be concluded from the data that the inability of the Ctrl peptide to formulate stable NPs is because natural alanines cannot provide enough hydrophobic interactions to condense the complexes into NPs. It is also noteworthy that hPep1, which has one octenyl‐ and one pentenyl‐alanine, and hPep2, which has two octenyl‐alanines, had similar siRNA release profiles despite only a three‐carbon atom difference. Overall, the hPep3 with three octenyl‐alanines had the highest stability.

Physicochemical properties of NPs such as size, size distribution [27], and zeta potential [28] affect their stability, biodistribution, pharmacokinetics, and clearance. These findings show that all tested hPep peptides can formulate siRNA into NPs with suitable characteristics for systemic use [29, 30]. While the NPs formulated with different hPeps are very similar in their physicochemical nature, their stability is strongly dependent on the hydrophobic interactions provided by the alkenyl‐alanines. As the helicity of the peptides also increases with hydrophobicity, we hypothesize that the helicity helps to further stabilize the complexes since it makes the peptide structure more rigid and stable [31, 32]. To determine if higher stability translates into better siRNA delivery efficiency, we continued with cell culture studies.

### 3.3 Octenyl‐Alanine Containing hPeps Display an Enhanced Cellular Uptake

**FIGURE 4:**
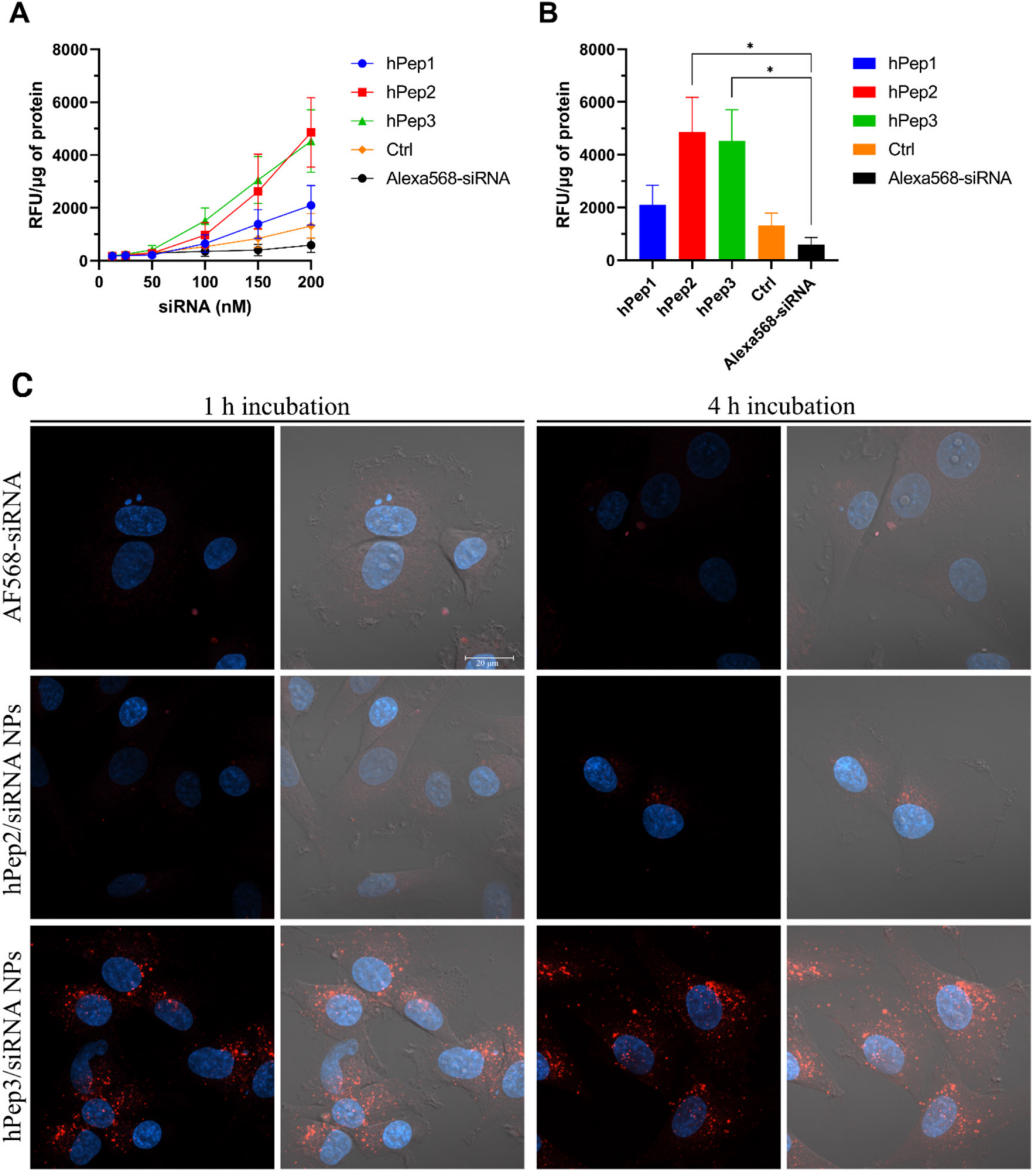
Cellular uptake efficiency and intracellular localization of hPep/siRNA NPs. For the uptake studies, the hPep/siRNA complexes were formed at MR30 with AlexaFluor568‐labeled siRNA. (A) Concentration‐dependent uptake of different hPep/siRNA NPs was studied in HEK‐Luc cells after 4 h treatment. (B) Snapshot of uptake levels at 200 nM siRNA concentration. All results were normalized to total protein content. The values represent the mean of at least three independent experiments performed in duplicates (mean ± SEM, *n* = 3). *p*‐values were determined by the one‐way ANOVA with Dunnett's multiple comparison test (ns—non‐significant, *p < 0.05, **p < 0.01, ***p < 0.001 and ****p < 0.0001). (C) Cellular localization of hPep2/AF568‐siRNA and hPep3/AF568‐siRNA NPs (red) was studied in U87‐Luc2 cells at 50 nM siRNA concentration by confocal microscopy. Cells were fixed 1 and 4 h after the treatments while Hoechst (blue) was used to stain the nuclei.

Efficient cellular uptake is a basic requirement for all oligonucleotide delivery systems. Hence, after confirming that all three hPep peptides form stable NPs with siRNA, we next sought to investigate how different alkenyl modifications in the hPeps affect the cellular uptake of their respective hPep/siRNA NPs. For this, HEK‐293 cells were treated with hPep/AF568‐siRNA NPs at different concentrations, and quantitative cellular uptake was measured. For all tested formulations, the cellular uptake was concentration dependent, with hPep2 and hPep3 NPs showing approximately twofold higher uptake than hPep1 (Figure 4A,B).

The quantitative uptake investigation was next complemented with intracellular distribution and localization studies. For that, we treated the U87‐Luc2 cells with hPep/AF568‐siRNA NPs at 50 nM siRNA and visualized their distribution with confocal microscopy at 1‐ and 4‐h time points. Surprisingly, as seen in Figure 4C, only hPep3 NPs were efficiently taken up by the cells. The hPep3/siRNA NPs showed strong punctuated fluorescent staining inside cells, indicating the involvement of endocytic transport mechanisms. Furthermore, the cellular uptake of hPep3/siRNA NPs was rapid, as already 1 h of treatment showed comparable uptake to 4 h. Uptake of hPep2 NPs was much lower, as only marginal intracellular signal was detected after 4‐h treatment.

Considering that the hydrophobicity of the hPeps is increasing in the order of hPep1 < hPep2 < hPep3 [22] and all of them form NPs with similar characteristics, we hypothesize that the uptake of hPep1 NPs is lower due to the lack of sufficient hydrophobic interactions with the cell membrane phospholipids, which could be further exacerbated by its weak amphipathic character. Given that the differences in the peptide hydrophobicity and amphipathicity come from the number of carbons introduced by the alanine and alkenyl‐alanine side chains: three carbons for Ctrl peptide, 13 and 17 for hPep1 and hPep2, and 24 for hPep3. This goes well in line with other similar findings [33], including our own [20], where we identified the N‐terminal fatty acid chain length of PepFect14 peptide as the key driver for cellular uptake—with uptake starting from 12 carbons and increasing with each added carbon up to 22. Although it might look from these findings that adding either of these modifications can enhance the uptake of any given CPP, our experience has shown that it is not that simple. Each peptide has its unique chemical properties and therefore, the modifications must be tailored individually, as for example, problems with solubility can arise with one but not with the other approach.

Together, these data show that increasing hydrophobicity (increasing number of carbons by increasing the number and length of alkenyl modifications) improves the uptake of the hPep/siRNA NPs to the cells.

### 3.4 Only hPep3 With Three Octenyl‐Alanines Enables Efficient Gene Silencing in Cell Cultures

After characterizing the cellular uptake of hPep/siRNA NPs, we next studied their ability to silence target gene expression in cell culture models. For that, we used modified reporter cell lines, HEK‐Luc and U87‐Luc2, that stably express the firefly luciferase enzyme where, upon successful siRNA delivery, the repression of luciferase expression can be quantified and correlated to functional gene silencing.

**FIGURE 5:**
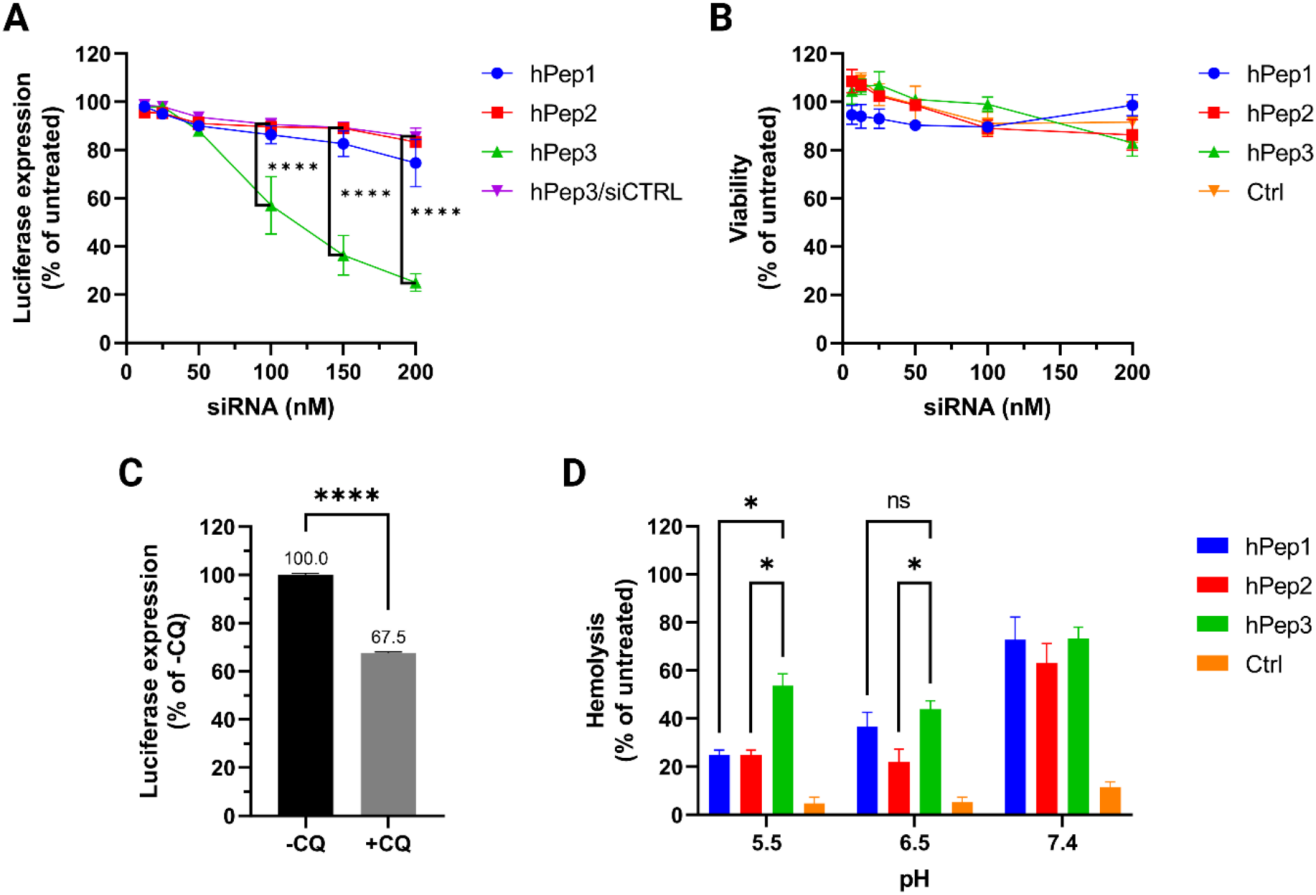
Pharmacological characterization of hPep/siRNA NPs in cell cultures. (A) Gene knockdown efficacy of hPep/siRNA NPs was studied in HEK‐Luc cells. Cells were treated with NPs (formulated at MR30) for 24 h at different siRNA concentrations. As a negative control, hPep3/siCTRL complexes were used. (B) Impact of hPep/siRNA NPs on cell viability and metabolic activity at different concentrations as evaluated by WST‐1 assay. (C) The biological activity of hPep3/siRNA NPs at 200 nM siRNA concentration with or without added CQ. (D) Erythrocyte leakage assay of RBCs treated with different hPep peptides at a concentration of 10 μM. The values represent the mean of at least three independent experiments done in duplicates (mean ± SEM, *n* = 3). HBG—HEPES buffered glucose. *p*‐values were determined by the two‐way ANOVA with Dunnett's multiple comparison test or with unpaired Student *t*‐test with Welch's correction in panel C (ns—non‐significant, *p < 0.05, **p < 0.01, ***p < 0.001, and ****p < 0.0001).

To quantify the ability of hPeps to mediate gene knockdown, we treated the cells with different hPep/siRNA NPs (MR30) over a range of concentrations. Out of all the tested peptides, only hPep3 with most alkenyl‐alanine modifications induced substantial RNAi‐mediated gene silencing after a 24‐h treatment. Starting from a concentration of 50 nM siRNA, a dose‐dependent gene silencing was achieved with up to 75% luciferase knockdown at 200 nM (Figure 5A). These results were further corroborated in another firefly luciferase‐encoding cell line, U87‐Luc2, where hPep3/siRNA NPs induced comparable or even higher levels of gene silencing as compared to HEK‐Luc cells (Figure S3). Interestingly, hPep2 was not able to induce any gene knockdown. We hypothesize it could be due to lower stability of hPep2/siRNA NPs (Figure 3C) and/or lower membrane activity of hPep2 at endolysosomal pH (Figure 5D) as the cellular uptake for hPep2 and hPep3 in this siRNA concentration range (100–200 nM) was comparable (Figure 4A). As expected, the silencing activity of hPep1 NPs remained on par with the level of negative control (hPep3/siCTRL) at all tested concentrations, coinciding well with its significantly lower cellular uptake efficacy, as shown in Figure 4. Longer treatment time (48 and 72 h) was not found to positively affect the silencing activity of hPep3 NPs (data not shown).

To see how gene silencing is affected by the peptide/siRNA molar ratio, an additional MR screening study was carried out in cells with hPep3 (Figure S3). The results showed that MR30 had the highest gene silencing efficiency out of all tested MRs that exhibit stable NPs (i.e., MR20–MR30, Figure 2B) thereby supporting its choice for further studies. Interestingly, this experiment also showed that the knockdown efficacy is not directly correlated to peptide amount in the complexes. A peak in knockdown efficacy was detected at MR12.5, followed by a decrease in efficacy up until MR20 and another increase up until MR30. We hypothesize that this jump in efficacy at MR12.5 occurs due to the formation of large peptide/siRNA aggregates (Figure 2B), which are more prone to sedimentation, thereby increasing the local concentration of the complexes in close proximity to the cells. Conclusively, these findings demonstrate the importance of conducting physicochemical and biological activity characterization studies while choosing optimal NP formulation conditions.

In addition to the pharmacological activity, the hPep/siRNA NPs were studied for their impact on cell viability. WST‐1 analysis showed that hPep/siRNA NPs did not decrease cell viability over 20% for any of the tested peptides, even at higher concentrations (Figure 5B). Together, these findings show that the luciferase knockdown efficacy of hPep3/siRNA NPs was not affected by cell viability and, most importantly, that all the tested peptides are safe to use.

### 3.5 Higher Membrane Activity at Endolysosomal pH Coincides With the Enhanced Activity of hPep3

Oligonucleotides gain access to the cells mostly by endocytosis. As they progress through the endo‐lysosomal system, the vast majority of the therapeutic material becomes stuck in the endosomes. Hence, endosomal release is widely considered the main rate‐limiting step in the effective delivery of oligonucleotides with all DDSs, including CPPs [11].

Our confocal microscopy studies (Figure 4C) indicated that hPep/siRNA NPs are likely taken up by the cells through endocytic uptake pathways. Therefore, we next wanted to understand to which extent the endosomal entrapment hinders their intracellular delivery. To test this, cells were treated with hPep3/siRNA NPs and exposed to CQ for endosomal release of the bioactive material. This approach allowed us to achieve about a 30% increase in luciferase knockdown compared to cells that did not receive CQ treatment (Figure 5C), as the luciferase expression dropped from 54.3% without CQ (−CQ) to 36.7% with CQ (+CQ) when normalized to untreated cells (Figure S4). To investigate this in more detail, we conducted a similar experiment with fluorescently labeled hPep3/AF568‐siRNA NPs and visualized the cells by confocal microscopy. Interestingly, after incubation with CQ, the endosomes containing NPs were visibly larger compared to endosomes without CQ treatment (Figure S5), corroborating that CQ causes endosomal swelling, which might be responsible for the enhanced release of endocytosed material. Together, these results show that hPep3/siRNA NPs are taken up by the cells through endocytic pathways and that, similarly to other delivery systems, including other CPPs, endosomal entrapment is a limiting factor in the silencing activity of the hPep peptides.

To shed light on why the hPep3 peptide, baring the most alkenyl‐alanine modifications, was the only hPep derivative that was able to induce effective gene silencing (Figure 5A), we next sought to study the membrane activity/endosomal release capacity of the hPep peptides in the ex vivo erythrocyte leakage assay [34]. In this assay, the erythrocytes are modeling the endosomal membrane and the lysis of the cell or release of hemoglobin resembles the rupture of endosomes. By varying the pH surrounding the erythrocytes, it is possible to mimic the endosomal release process from early (pH 6.5) to late endosomes (pH 5.5) and to evaluate the endosomolytic properties of hPep peptides at different stages of endocytosis. When the membrane activity of hPeps alone was compared at pH 7.4, mimicking the conditions at the plasma membrane, all hPeps showed a similar 70%–80% hemolytic activity, with only the Ctrl peptide remaining at around 10% of hemolysis (Figure 5D). At pH 6.5, an overall decrease in hemolytic activity could be detected. Interestingly, at pH 5.5, hPep3 was able to maintain significantly higher lytic activity (~50%) than hPep1 and hPep2 (both ~25%), while the Ctrl peptide without the alkenyl‐alanine modifications was showing almost no activity. We hypothesize that the difference in membrane activity at lower pHs, together with its higher helical content, could be the possible reasons why hPep3/siRNA NPs are biologically active while hPep2 NPs, which are also effectively taken up by the cells (Figure 4) remain inactive (Figure 5A). Taken together, the erythrocyte leakage data clearly indicates that alkenyl‐alanines are responsible for the membrane activity of hPep peptides when compared to the Ctrl peptide with natural alanines, which had very limited membrane activity. Furthermore, the higher number of octenyl‐alanines seems to be beneficial for providing membrane activity at lower pHs, i.e., mimicking endo‐lysosomal conditions, and this is likely the key factor in the siRNA delivery efficiency of hPep3 over the other hPeps.

In conclusion, out of all the tested peptides, only the most hydrophobic and amphipathic hPep analog, hPep3, induced both a high level of cellular uptake, endosomolytic activity, and effective RNAi‐mediated gene silencing in cellular model systems. Based on this, further studies were focused only on hPep3.

### 3.6 Knockdown of an Endogenous Target Gene (CD45) With hPep3/siRNA NPs

After confirming that the hPep/siRNA NPs can induce effective gene silencing in reporter model systems, we next set out to validate its gene silencing potential against an endogenous target gene. This work was carried out on CD45, a transmembrane protein tyrosine phosphatase, which is a common leukocyte antigen [35] associated with a variety of diseases including leukemia [36], lymphoma [37] and several autoimmune conditions [38, 39]. As CD45 is constitutively highly expressed on all cells of hematopoietic origin, it serves as an excellent endogenous target and has often been used to evaluate the potency of DDSs [40, 41, 42].

**FIGURE 6:**
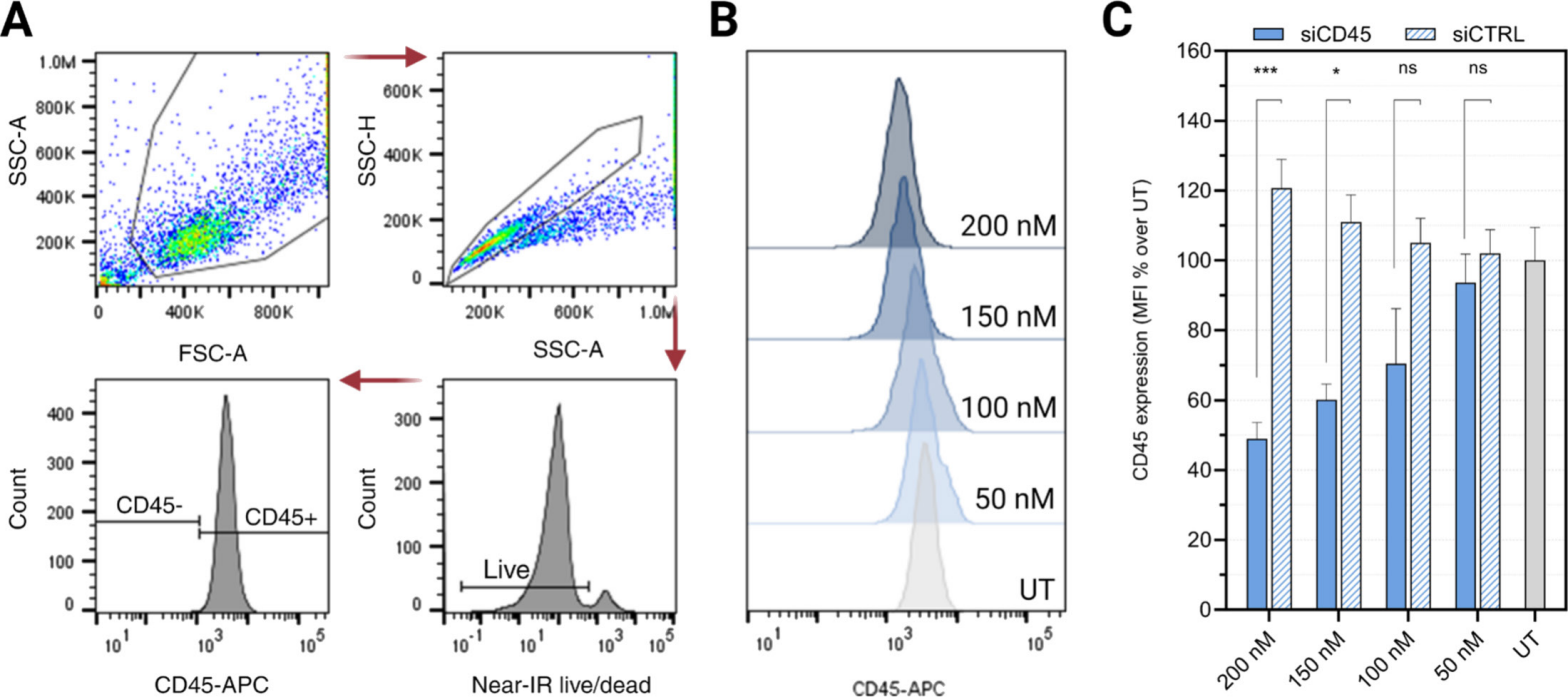
CD45 silencing in RAW 264.7 macrophages. (A) Flow cytometry gating strategy. (B) Representative image of the concentration‐dependent silencing of endogenous CD45 target at a siCD45 concentration of 50–200 nM. (C) Median fluorescence intensity (MFI) of cells treated with hPep3/siCD45 NPs or hPep3/siCTRL NPs formulated at MR30. UT—untreated cells. Results are shown as mean ± SEM, *n* = 3. *p*‐values were determined by the two‐way ANOVA with Šidák's multiple comparison test (ns—non‐significant, *p < 0.05, **p < 0.01, ***p < 0.001, and ****p < 0.0001).

To test the endogenous CD45 gene silencing potential, RAW 264.7 murine macrophages were treated with hPep3/siRNA NPs for 24 h, followed by flow cytometry analysis of CD45 expression (Figure 6A). As seen in Figure 6B,C, treatments with hPep3/siRNA NPs induced a concentration‐dependent CD45 protein downregulation, exerting more than 50% knockdown of CD45 protein expression at a siRNA concentration of 100 nM. The efficacy of hPep3 was comparable to the effects seen in the luciferase reporter system and shows the potential of hPep3 to achieve effective RNAi‐mediated gene silencing of the endogenous target.

### 3.7 hPep3 Increases the Accumulation of siRNA in the Lungs, Liver, and Spleen

**FIGURE 7:**
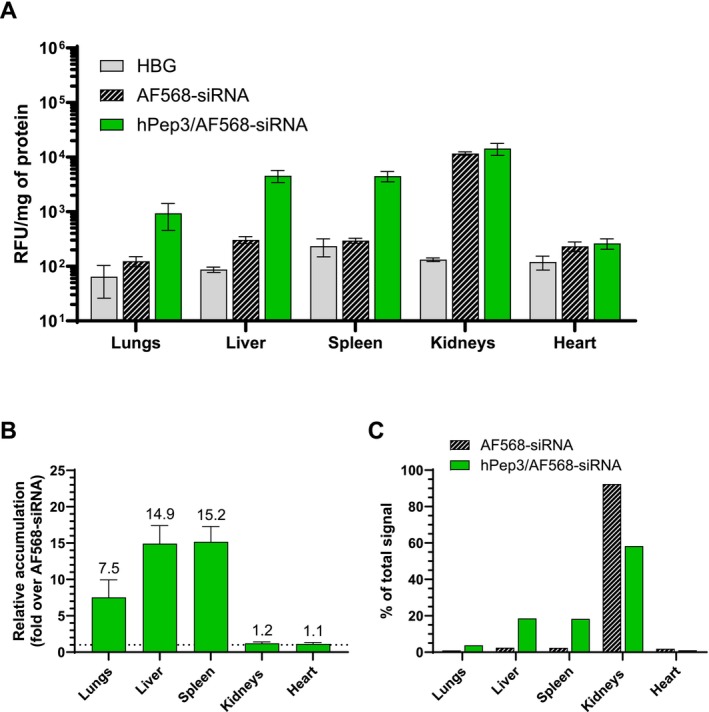
In vivo biodistribution of hPep3/siRNA NPs at MR30. (A) BALB/c mice were intravenously injected with AlexaFluor568‐siRNA (black), hPep3/AlexaFluor568‐siRNA NPs (green) at siRNA dose 1 mg/kg and with vehicle control, HEPES‐buffered glucose (HBG) (gray) (*n* = 3 animals per group). Four hours after the injection tissues were dissected and AlexaFluor568 fluorescence was quantified from homogenized tissues. (B) The effect of hPep3 on siRNA accumulation presented as fold increase over free AlexaFluor568‐siRNA. (C) Tissue distribution as a fraction of the total accumulated fluorescence signal from all measured organs. The values represent the mean ± SEM (*n* = 3 per group).

Despite the advances in siRNA delivery, targeting siRNAs to the extra‐hepatic tissues is still a major problem. After demonstrating that hPep3/siRNA NPs can effectively deliver siRNA in several in vitro models, we sought to understand if hPep3 could also improve the delivery of siRNA in in vivo settings. For this, mice were injected with AlexaFluor568‐labeled siRNA (AF568‐siRNA) either nakedly or in formulation with hPep3 (hPep3/AF568‐siRNA). Four hour post i.v. injection, the tissues were harvested, and fluorescence was quantified from tissue lysates. The biodistribution study indicated that naked AF568‐siRNA accumulates primarily in kidneys (> 90% of detected signal), while its accumulation in other tissues is very limited (Figure 7A,C). In animals treated with hPep3/siRNA NPs, siRNA delivery was increased in several tissues, illustrated by the 7.5‐, 14.9‐, and 15.2‐fold increase in fluorescence in lungs, liver, and spleen when compared to naked siRNA at 1 mg/kg, respectively (Figure 7B). When looking at the signal distribution between the organs, it was evident that encapsulation with hPep3 decreased siRNA accumulation in the kidneys by ~30% and concomitantly resulted in an increased nucleic acid delivery to the liver, spleen, and lungs (Figure 7C). Moreover, the total siRNA accumulation across tissues was increased with hPep3/siRNA NPs by about 1.9‐fold compared to free siRNA (data not shown).

Overall, the hPep3/siRNA NPs were well tolerated and facilitated increased accumulation of siRNA in a number of tissues, including extra‐hepatic sites, demonstrating the potential of employing alkenyl‐alanine modifications for targeting organs outside the liver.

## 4 Conclusions

Therapeutics based on the powerful gene silencing method of RNAi have made their transition to the clinics for a number of liver‐specific diseases. This success can mainly be attributed to the development of effective drug delivery technologies that can specifically target hepatocytes. However, access to extra‐hepatic tissues still remains a major hurdle. To overcome this, the development of more effective drug delivery technologies with wider biodistribution and activity profiles than those with presently available technologies is required. Here, we demonstrate the following beneficial effects of incorporating alkenyl‐alanines into the structure of hPep peptides. By providing extra hydrophobic interactions, helicity, and amphipathicity, they help to promote encapsulation of siRNA into more stable NPs, achieve membrane activity necessary for enhanced cellular uptake and endosomal escape, and gain gene silencing on artificial and endogenous gene targets in vitro. Furthermore, employing such modifications improves the in vivo delivery of hPep/siRNA NPs in several tissues, including extra‐hepatic sites. Altogether, the alkenyl‐alanine modifications offer a simple and robust means for gaining biologically active NPs and serve as an alternative to the fatty acid conjugation approach for developing effective carriers for RNA.

## Author Contributions

Conceptualization, T.L. (Tõnis Lehto), S.E.A., T.L. (Taavi Lehto).

Data curation, M.I., H.S., A.L., T.L. (Tõnis Lehto).

Formal analysis, M.I., H.S., A.L., T.L. (Tõnis Lehto).

Funding acquisition, S.E.A. and T.L. (Taavi Lehto).

Investigation, M.I., H.S., O.P.B.W., A.L., S.B., T.L. (Tõnis Lehto).

Methodology, M.I., H.S., O.P.B.W., A.L., S.B., T.L. (Tõnis Lehto).

Project administration, S.E.A. and T.L. (Taavi Lehto).

Supervision, S.E.A. and T.L. (Taavi Lehto).

Visualization, M.I., H.S., A.L., T.L. (Tõnis Lehto).

Writing – original draft, M.I., T.L. (Tõnis Lehto).

Writing – review and editing, M.I., T.L. (Tõnis Lehto), H.S., A.L., S.E.A., O.P.B.W., T.L. (Taavi Lehto).

All authors have read and agreed to the published version of the manuscript.

## Ethics Statement

The animal experiments were carried out in accordance with the Guidelines for Care and Use of Laboratory Animals of the University of Tartu and were approved by the Estonian Laboratory Animal Ethics Committee (approval no 185, dated Feb 16, 2021).

## Consent

The authors have nothing to report.

## Conflicts of Interest

O.P.B.W. has equity interest in Evox Therapeutics. S.E.A. is a co‐founder and shareholder in Evox Therapeutics. Other authors declare no conflicts of interest.

## Supporting information


**Figure S1:** Size and zeta potential of hPep/siRNA complexes at different molar ratios. DLS was used to measure size (blue) and zeta potential (red). The values represent the mean of at least three independent experiments (mean ± SEM, *n* = 3).
**Figure S2:** Size distribution graphs of hPep/siRNA NPs at MR30. DLS was used to measure the size distribution of NPs. The curves represent the mean of at least three independent experiments, and the data correspond to the values presented in Figure 2A.
**Figure S3:** Evaluation of hPep3/siRNA MR on gene silencing in U87‐Luc2 cells. Complexes formulated over a range of MRs and cells were treated for 24 h at different siLuc2 concentrations. The values represent the mean of at least three independent experiments (mean ± SEM, *n* = 3).
**Figure S4:** Chloroquine enhances the silencing activity of hPep3/siRNA NPs in HEK‐Luc cells. Complexes were formulated at MR30 and cells treated at a concentration of 200 nM siRNA. The values represent the mean of at least three independent experiments (mean ± SEM, *n* = 3). *p*‐values were determined by unpaired Student *t*‐test with Welch's correction (ns—non‐significant, *p < 0.05, **p < 0.01, ***p < 0.001 and ****p < 0.0001).
**Figure S5:** Chloroquine treatment induces enlargement of endosomes. HEK‐Luc cells were treated with hPep3/siRNA NPs formulated at MR30 at a concentration of 50 nM siRNA for 4 h followed by 2‐h treatment with fresh media or media containing 50 μM of chloroquine.

## Data Availability

All relevant data are available within the article and its Supplementary Information files or from the corresponding authors upon reasonable request.
