# Supplementary material for: Amphipathic Octenyl‐Alanine Modified Peptides Mediate Effective siRNA Delivery
Source: J Pept Sci. 2025 Sep 7;31(10):e70054. doi: 10.1002/psc.70054 (PMC12414742; doi:10.1002/psc.70054)
Supplement: Supplementary file 1 — Figure S1: Size and zeta potential of hPep/siRNA complexes at different molar ratios. DLS was used to measure size (blue) and zeta potential (red). The values represent the mean of at least three independent experiments (mean ± SEM, n = 3). Figure S2: Size distribution graphs of hPep/siRNA NPs at MR30. DLS was used to measure the size distribution of NPs. The curves represent the mean of at least three independent experiments, and the data correspond to the values presented in Figure 2A. Figure S3: Evaluation of hPep3/siRNA MR on gene silencing in U87‐Luc2 cells. Complexes formulated over a range of MRs and cells were treated for 24 h at different siLuc2 concentrations. The values represent the mean of at least three independent experiments (mean ± SEM, n = 3). Figure S4: Chloroquine enhances the silencing activity of hPep3/siRNA NPs in HEK‐Luc cells. Complexes were formulated at MR30 and cells treated at a concentration of 200 nM siRNA. The values represent the mean of at least three independent experiments (mean ± SEM, n = 3). p‐values were determined by unpaired Student t‐test with Welch's correction (ns—non‐significant, *p < 0.05, **p < 0.01, ***p < 0.001 and ****p < 0.0001). Figure S5: Chloroquine treatment induces enlargement of endosomes. HEK‐Luc cells were treated with hPep3/siRNA NPs formulated at MR30 at a concentration of 50 nM siRNA for 4 h followed by 2‐h treatment with fresh media or media containing 50 μM of chloroquine. [file PSC-31-e70054-s001.docx]

**Supplementary Information**





**Supplementary Figure 1. Size and zeta potential of hPep/siRNA complexes at different molar ratios.** DLS was used to measure size (blue) and zeta potential (red). The values represent the mean of at least three independent experiments (mean ± SEM, n = 3).

**Supplementary Figure 2. Size distribution graphs of hPep/siRNA NPs at MR30.** DLS was used to measure the size distribution of NPs. The curves represent the mean of at least three independent experiments and the data corresponds to the values presented in Figure 2A.

**Supplementary Figure 3. Evaluation of hPep3/siRNA MR on gene silencing in U87-Luc2 cells.** Complexes formulated over a range of MRs and cells were treated for 24 h at different siLuc2 concentrations. The values represent the mean of at least three independent experiments (mean ± SEM, n = 3).

**Supplementary Figure 4. Chloroquine enhances the silencing activity of hPep3/siRNA NPs in HEK-Luc cells.** Complexes were formulated at MR30 and cells treated at 200 nM siRNA concentration. The values represent the mean of at least three independent experiments (mean ± SEM, n = 3). *p*-values were determined by unpaired student t-test with Welch’s correction (ns—non-significant, * p < 0.05, ** p < 0.01, *** p < 0.001 and **** p < 0.0001).


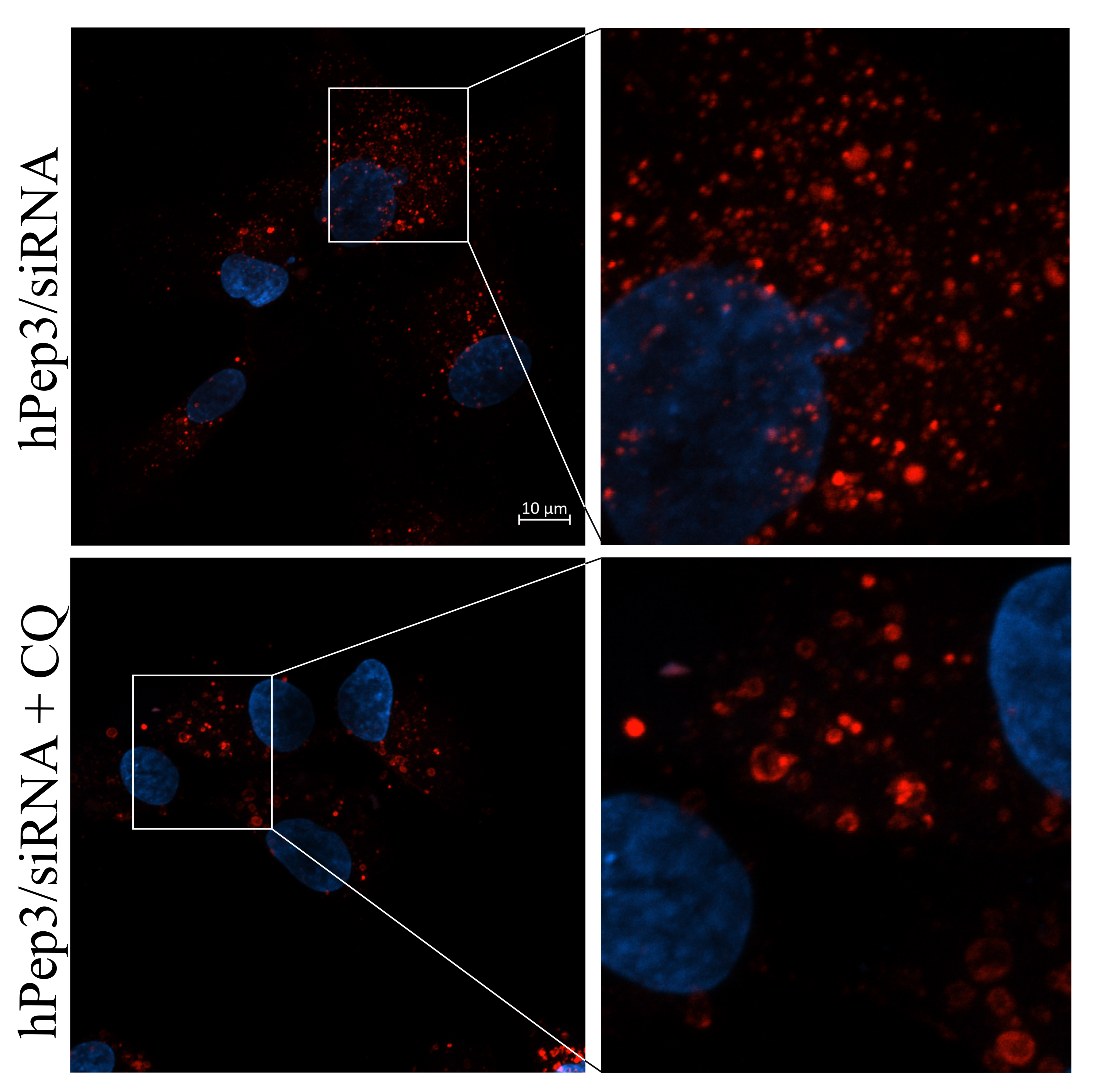


**Supplementary Figure 5. Chloroquine treatment induces enlargement of endosomes.** HEK-Luc cells were treated with hPep3/siRNA NPs formulated at MR30 at 50 nM siRNA concentration for 4 h followed by 2 h treatment with fresh media or media containing 50 µM chloroquine.
